# Regulated Mucin Secretion from Airway Epithelial Cells

**DOI:** 10.3389/fendo.2013.00129

**Published:** 2013-09-18

**Authors:** Kenneth B. Adler, Michael J. Tuvim, Burton F. Dickey

**Affiliations:** ^1^Department of Molecular Biomedical Sciences, North Carolina State University College of Veterinary Medicine, Raleigh, NC, USA; ^2^Department of Pulmonary Medicine, The University of Texas MD Anderson Cancer Center, Houston, TX, USA

**Keywords:** secretion, exocytosis, mucin, mucus, MARCKS, Munc18, Munc13, synaptotagmin

## Abstract

Secretory epithelial cells of the proximal airways synthesize and secrete gel-forming polymeric mucins. The secreted mucins adsorb water to form mucus that is propelled by neighboring ciliated cells, providing a mobile barrier which removes inhaled particles and pathogens from the lungs. Several features of the intracellular trafficking of mucins make the airway secretory cell an interesting comparator for the cell biology of regulated exocytosis. Polymeric mucins are exceedingly large molecules (up to 3 × 10^6^ Da per monomer) whose folding and initial polymerization in the ER requires the protein disulfide isomerase Agr2. In the Golgi, mucins further polymerize to form chains and possibly branched networks comprising more than 20 monomers. The large size of mucin polymers imposes constraints on their packaging into transport vesicles along the secretory pathway. Sugar side chains account for >70% of the mass of mucins, and their attachment to the protein core by O-glycosylation occurs in the Golgi. Mature polymeric mucins are stored in large secretory granules ∼1 μm in diameter. These are translocated to the apical membrane to be positioned for exocytosis by cooperative interactions among myristoylated alanine-rich C kinase substrate, cysteine string protein, heat shock protein 70, and the cytoskeleton. Mucin granules undergo exocytic fusion with the plasma membrane at a low basal rate and a high stimulated rate. Both rates are mediated by a regulated exocytic mechanism as indicated by phenotypes in both basal and stimulated secretion in mice lacking Munc13-2, a sensor of the second messengers calcium and diacylglycerol (DAG). Basal secretion is induced by low levels of activation of P_2_Y_2_ purinergic and A3 adenosine receptors by extracellular ATP released in paracrine fashion and its metabolite adenosine. Stimulated secretion is induced by high levels of the same ligands, and possibly by inflammatory mediators as well. Activated receptors are coupled to phospholipase C by Gq, resulting in the generation of DAG and of IP_3_ that releases calcium from apical ER. Stimulated secretion requires activation of the low affinity calcium sensor Synaptotagmin-2, while a corresponding high affinity calcium sensor in basal secretion is not known. The core exocytic machinery is comprised of the SNARE proteins VAMP8, SNAP23, and an unknown Syntaxin protein, together with the scaffolding protein Munc18b. Common and distinct features of this exocytic system in comparison to neuroendocrine cells and neurons are highlighted.

## Biology and Pathophysiology of Airway Mucus

Mucus has physical characteristics on the border between a viscous fluid and a soft and elastic solid (1). These characteristics are conferred by a semi-dilute network of polymerized mucins in water. The secreted, polymeric mucins expressed in the airways are Muc5ac and Muc5b (2, 3). (Note, lower case letters are used to designate non-human mammalian mucins, while MUC5AC and MUC5B designate the human orthologs. In this review, we use lower case letters to designate all mammalian mucins, and only use upper case letters when referring specifically to human data.) In healthy airway mucus, water accounts for about 98% of the mass, mucins for about 0.7%, and salts and small amounts of other macromolecules for the rest. The mucus layer lies atop a denser periciliary layer containing membrane-tethered glycoconjugates, including glycosaminoglycans and membrane-spanning mucins (Muc 1, 4, and 16) (4–6). The mucus layer is continually swept from distal to proximal airways by beating cilia, and is eventually propelled out of the lungs into the pharynx and swallowed, removing entrapped particles, pathogens, and dissolved chemicals. The critical importance of the mucus layer in airway defense is shown by the spontaneous inflammatory lung and nasal disease that develops in mice in which the constitutively produced mucin, Muc5b, has been deleted (1).

**Figure 1:**
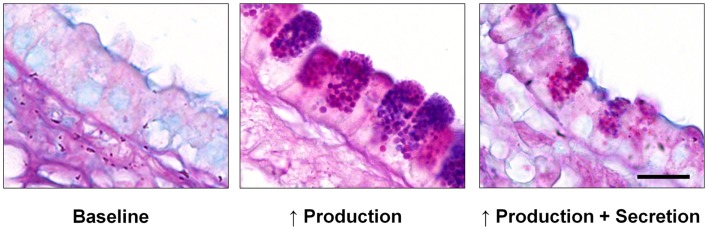
**Mucin production and secretion in the mouse airway**. Left – In the healthy baseline state, alternating ciliated and domed secretory cells are seen, with no mucin granules visible by Alcian blue and periodic acid Schiff (AB-PAS) staining. Center – Numerous large mucin granules are visible in secretory cells 3 days after mucin production is increased by IL-13-dependent allergic inflammation as described (51). Right – Exocytic secretion of the intraepithelial mucin stored in inflamed airway epithelium induced by brief exposure to an ATP aerosol as described (51). Scale bar is 10 μm.

In order to replenish the mucus layer, mucins are continuously synthesized and released by secretory cells that form a mosaic with ciliated cells, with similar numbers of both cell types (Figure 1, left). In allergic lung inflammation, which appears to be a parasitic defense gone awry (4, 7), the second polymeric airway mucin, Muc5ac, is produced in large quantities (Figure 1, center and right). Whereas increased mucin production alone does not appear to lead to pathology (8), the production of large amounts of mucin together with its rapid secretion (“mucus hypersecretion”) can overwhelm available liquid resulting in formation of excessively viscoelastic mucus that is poorly cleared by ciliary action or cough. When coupled with airway narrowing due to bronchoconstriction in asthma, this can lead to widespread airway closure with serious consequences. Mucus hypersecretion is also an important feature of chronic obstructive pulmonary disease (COPD), cystic fibrosis (CF), and idiopathic bronchiectasis (1).

A key event in mucus secretion is its hydration immediately after exocytosis (4, 9, 10). Mucins are packaged dehydrated in secretory granules, and must adsorb more than 100-fold their mass of water soon after secretion in order to attain the appropriate viscoelasticity for ciliary clearance. Water in the airway lumen is controlled both by the release of chloride through CFTR, CaCC, and Slc26A9, and by the absorption of sodium by ENaC, with water passively following the flux of ions (11–13). Coupling of the secretion of chloride and mucins is accomplished by paracrine signaling through ATP, adenosine, and other extracellular signaling molecules. In CF, mutation of the principal chloride channel, CFTR, results in insufficient luminal water that causes the formation of underhydrated mucus which is excessively viscoelastic and difficult to clear. The underhydration is exacerbated because CFTR is also a channel for bicarbonate, which is needed to chelate calcium (9, 14). In acidic secretory granules of the intestinal epithelium, calcium binds to the N-terminus of MUC2, which is the secreted mucin most similar to MUC5AC in structure, and organizes the mucin in such a way that it can be secreted without entanglement (15). Too little bicarbonate at mucin release prevents normal mucin unfolding and leads to formation of abnormally dense mucus (16); similar mechanisms likely operate in the airway.

In summary, the mucus layer forms a mobile, essential barrier that protects the lungs when it is functioning properly, but dysfunction of the mucus layer plays a prominent role in all of the common diseases of the airways.

## Mucin Synthesis, Processing, and Packaging

Muc5b/MUC5B is transcribed constitutively throughout the conducting airways from the trachea down to but not including terminal bronchioles (1, 17, 18). Muc5ac is produced in low amounts or not at all in healthy mice, although in humans MUC5AC is produced constitutively in proximal airways (trachea and bronchi) (1). In allergic inflammation the production of Muc5ac increases dramatically (40- to 200-fold) in the airways of mice and in cultured human airway epithelial cells (19–21). Both mucins are produced in the same secretory cells, but even in conditions of severe inflammation they are not produced in small airways, which makes teleologic sense in that their small luminal diameters (<200 μm) make them highly susceptible to occlusion.

After translation at the ER, Muc5ac, and Muc5b undergo initial polymerization as homodimers (3). These are among the largest macromolecules encoded in the mammalian genome, and their processing induces a stress response in the ER (22). Proper folding and polymerization require the protein disulfide isomerase Agr2, whose deletion in mice results in absent intestinal mucin (23) and in reduced airway mucin in the setting of allergic lung inflammation (24). The transport of polymeric mucins from the ER to the Golgi and through the Golgi has been little studied, but likely involves modulation of COP-II transport vesicle size to accommodate large cargoes as has been described for collagen (25). In the Golgi, Muc5b undergoes further polymerization in linear chains up to 20 monomers in length (3, 26). The structure of Muc5ac is less well studied, but appears to be more similar to Muc2 that forms branched polymers resulting in formation of a covalent net (9). Both mucins undergo O-glycosylation in the Golgi that result in mature glycoproteins that are more than 70% carbohydrate with a general negative charge due to sulfation or sialylation of many terminal sugars (27).

Export of polymeric mucins from the trans-Golgi and lateral fusion of post-Golgi vesicles to form secretory granules are additional transport steps that have been poorly studied. Similar to the case of COP-II vesicles, post-Golgi clathrin-coated vesicles have recently been shown to be capable of size variation to accommodate large cargo proteins (28). It is probable this mechanism is also utilized for mammalian mucins because, in fruit flies, assembly of large salivary mucin granules requires clathrin and the adaptor AP-1 (29). Mature mucin secretory granules are very large, with a mean diameter of 1 μm. Their exocytic fusion is highly regulated by extracellular secretagogues (Table 1), as described below. It should be noted that even though additional secretory pathways have been described in other cell types, such as a minor regulated pathway for secretion of immature granules and the compound exocytosis of mature granules (30), these are not well-described in airway secretory cells and will not be addressed in this review.

**Table 1:** **Ligands shown to induce mucin secretion**.

Ligand	Receptor; site of action	Reference
ATP, UTP	P_2_Y_2_; epithelium	Chen et al. (68), Danahay et al. (49), Ehre et al. (37), Kemp et al. (69), and Kim and Lee (70)
Adenosine	A3AR; epithelium (in mice but not humans, dogs, or guinea pigs)	Young et al. (62)
Proteases	PAR1, PAR2, other; epithelium	Breuer et al. (71), Jones et al. (47), Liu et al. (72), and Park et al. (39)
Acetylcholine	Unknown; may be indirect	Singer et al. (34)
Histamine	Unknown; may be indirect	Huang et al. (73)
Serotonin	Unknown; may be indirect	Foster et al. (36)
Capsaicin (substance P)	NK1; may be indirect	Guo et al. (74) and Kuo et al. (75)
Ionomycin (calcium)	Syt2, Munc13, PKC, other; epithelium (intracellular)	Danahay et al. (49), Ehre et al. (37), Tuvim et al. (46), and Zhu et al. (18)
PMA	Munc13-2, PKC; epithelium (intracellular)	Danahay et al. (49), Ehre et al. (37), and Zhu et al. (18)

In the left column are ligands reported to induce secretion of mucin from airway surface epithelial cells. In the middle column are receptors for these ligands and whether they act directly on epithelial cells. In the right column are selected references that offer evidence for the activity of the ligands, their receptors, and their cellular localization. The first three rows (white background) show ligands that appear to act directly on epithelial cell surface receptors based upon in vitro and/or in vivo studies; the next four rows (gray background) show ligands that generally act on cell surface receptors, but may activate cells in the airway other than epithelial cells that in turn activate epithelial cells; the last two rows show ligands that act on intracellular targets. PMA, phorbol 12-myristate 13-acetate.

## Mucin Granule Positioning for Secretion

The movement of mature mucin granules to the plasma membrane for exocytosis has been the subject of work in numerous laboratories for many years. In the 1990s, work in the Adler laboratory turned to the Myristoylated Alanine-Rich C Kinase Substrate (MARCKS) protein. MARCKS was a known actin-binding protein and protein kinase C (PKC) substrate (31), and it was known that PKC activation enhanced mucin secretion (32), so MARCKS was a logical candidate regulator of mucin granule movement.

MARCKS is a rod-shaped 87 kDa protein that is ubiquitously expressed. Three domains of MARCKS are conserved evolutionarily. First is the Phosphorylation Site Domain (PSD), also known as the “effector domain,” a highly basic 25 amino acid stretch containing a number of serine residues that are phosphorylated by PKC. This domain also binds calcium/calmodulin and crosslinks actin filaments. Second is the Multiple Homology (MH2) domain, whose function is unknown. Third is the N-terminal region containing 24 amino acids and a myristic acid moiety involved in binding to membranes. MARCKS knockout mice die at birth or soon afterward, so peptides that might compete with native MARCKS to inhibit its function were generated by the Adler laboratory in collaboration with the Blackshear laboratory. These were tested using normal human bronchial epithelial (NHBE) cells grown in air-liquid interface culture to maintain their well-differentiated state. Peptides identical to the PSD site tended to induce a toxic response, but a peptide identical to the N-terminus had a strong inhibitory effect on mucin secretion induced by a combination of phorbol ester, a PKC activator, and 8-bromo-cyclic GMP, a protein kinase G (PKG) activator (33), or by the more physiologically relevant stimulus UTP. This peptide was named Myristoylated N-terminal Sequence (MANS), and a control missense peptide was named Random N-terminal Sequence (RNS). In contrast to MANS, RNS was without effect on mucin secretion. Additional studies showed that MARCKS phosphorylation in response to protein kinase activation, followed by dephosphorylation catalyzed by a protein phosphatase type 2A (PP2A), were critical to MARCKS function. This was the first publication to show a specific biological function for MARCKS, and suggested a mechanism whereby MARCKS came off the inner face of the plasma membrane when phosphorylated by PKC, then bound to mucin granules at the N-terminus and the cytoskeleton at the PSD site, serving as a bridge for granule transport to the plasma membrane by the cytoskeleton (33).

To examine the function of MARCKS *in vivo*, mice with mucous metaplasia induced by allergic inflammation (see Mucin Exocytosis, below) were then exposed to aerosolized methacholine to induce mucin secretion. Intratracheal pretreatment with MANS dose-dependently inhibited mucin secretion (34), and it attenuated airflow obstruction about 40% (35). Gold-labeling of stimulated cells revealed MARCKS to be morphologically associated with mucin granules, and treatment with MANS but not RNS blocked the association (34). Additional studies performed in mice with human neutrophil elastase instilled in the airways to induce mucous metaplasia showed similar results, with MANS but not RNS attenuating both mucin secretion and airway hyperreactivity in response to serotonin (36).

Subsequent studies have revealed that PKC δ and ε isoforms are involved in stimulated mucin secretion (37, 38), and that PKCδ-provoked secretion depends on phosphorylation of MARCKS (38, 39). Another question was the mechanism of translocation of MARCKS from the plasma membrane to mucin granules. Co-immunoprecipitation studies revealed an association between MARCKS and two previously described chaperones – Heat Shock Protein 70 (HSP70) and Cysteine String Protein (CSP) (40). Of interest, there was previously known to be direct and specific interaction of HSP70 with CSP (41). Western blotting and proteomic analysis of mucin granule membranes, ultrastructural immunohistochemistry, and immunoprecipitation experiments showed that MARCKS, HSP70, and CSP form a trimeric complex associated with the granule membrane (42, 43). Functional studies in a bronchial epithelial cell line using siRNA to knock down expression of MARCKS, HSP70, or CSP resulted in the attenuation of stimulated mucin secretion (42).

Additional studies have examined interactions among MARCKS, the chaperones, and cytoskeletal proteins. Treatment of NHBE cells with the pyrimidinone MAL3-101, an HSP70 inhibitor, or siRNA against HSP70, attenuated phorbol ester-stimulated mucin secretion, and blocked trafficking of fluorescent-tagged MARCKS (42, 44). In preliminary studies, cell-permeant peptides that target different domains of CSP were utilized to show that the C-terminus of CSP, rather than the more frequently studied “J” domain, appears to be involved in attachment of MARCKS to mucin granule membranes and resultant secretion. MARCKS has been found to bind both actin and myosin (33), and recent experiments showed that the myosin family involved is Myosin V (45). A possible contributing mechanism of MARCKS action besides granule transport could be the remodeling of apical actin (30). Exocytic Rab GTPases of the 3 and 27 subfamilies interact with the cytoskeleton and catalyze loose tethering of secretory granules to the plasma membrane in other cell types; Rab3D and Rab27A are expressed in airway secretory cells (30, 46), though they have not yet been functionally implicated in mucin secretion or MARCKS interaction. Another possibly important interaction is with VAMP8 that has been identified as the principal t-SNARE in mucin secretion (47) (see Core Exocytic Machinery, below). Preliminary studies from the Adler laboratory show that MARCKS and CSP bind VAMP8 on mucin granules. In summary, MARCKS engages in multiple protein interactions that together help position mucin secretory granules for exocytotic release. For a listing of proteins known to localize to the mucin granule membrane, see Table 2.

**Table 2:** **Proteins associated with airway epithelial mucin granules**.

Protein	Reference
ClCa3	Leverkoehne and Gruber (76), Lin et al. (45), Park et al. (40), Raiford et al. (43), and Singer et al. (34)
CFTR	Lesimple et al. (77)
CSP	Fang et al. (44), Lin et al. (45), Park et al. (40), and Raiford et al. (43)
HSP70	Fang et al. (44), Lin et al. (45), Park et al. (40), and Raiford et al. (43)
MARCKS	Fang et al. (44), Li et al. (33), Lin et al. (45), Park et al. (40), Park et al. (38), Raiford et al. (43), and Singer et al. (34)
Myosin V	Lin et al. (45) and Raiford et al. (43)
Rab3D	Evans et al. (51) and Tuvim et al. (46)
Syt2	Tuvim et al. (46)
VAMP8	Jones et al. (47)
VNUT	Sesma et al. (78)

Proteins that have been found to be associated with mucin granules of airway surface epithelial cells are listed alphabetically in the first column, and references for the association are reported in the second column. See Table 1 in Ref. (43) for a full listing of all proteins found by LC/MS to associate with mucin granules, though not all of these have been validated. ClCa, calcium-activated chloride channel; CFTR, cystic fibrosis transmembrane conductance regulator; CSP, cysteine string protein; HSP, heat shock protein; VNUT, vesicular nucleotide transporter.

## Mucin Exocytosis

Mucins are secreted into the airway lumen at a low basal rate and a high stimulated rate (1, 30). It is difficult to precisely define the difference in these rates because their measurement depends upon intracellular mucin content, the time interval of observation, and the post-exocytic release of mucins and their maturation to mucus for most assays. Despite these limitations, the rate of stimulated secretion has been generally found to exceed the rate of basal secretion by ∼5-fold over durations of 1 h or less by a variety of techniques (18, 37, 48, 49). The basal rate of secretion matches the basal rate of mucin synthesis in the distal airways of humans and all the airways of mice so that there is little intracellular mucin accumulation in the healthy state (Figure 1, left). Small amounts of intracellular mucin in this setting can be detected by sensitive immunohistochemical techniques that involve signal amplification (18, 50), but generally are not detectable by histochemical stains (51). The proximal airways of humans do contain histochemically apparent mucin associated with the constitutive expression of MUC5AC (1), but most functional studies of the exocytic machinery have been performed in mouse models so these will be the focus of further discussion. For comparison with *in vitro* systems studied by electrophysiologic (49) and videomicroscopic (52) techniques, the reader is referred to the referenced articles.

A regulated exocytic mechanism mediates both basal and stimulated mucin secretion as indicated by abnormal phenotypes in both basal and stimulated secretion when Munc13-2, a sensor of second messengers (see Extracellular Signaling and the Exocytic Regulatory Machinery), is deleted in mice (18). A defect in basal mucin secretion can be detected as the spontaneous accumulation of intracellular mucin in the absence of increased mucin synthesis (53). To measure stimulated secretion, it is useful to first induce increased mucin production and accumulation (mucous metaplasia) with allergic inflammation (Figure 1, center), such as by IL-13 instillation or ovalbumin immunization and challenge (51, 53). A defect in stimulated mucin secretion can then be detected as the failure to release intracellular mucin in response to a strong agonist such as ATP (Figure 1, right). Differential effects of the deletion of genes encoding various exocytic proteins on basal and stimulated mucin secretion indicate which proteins participate in which secretory state. In general, deletion of components of the core exocytic machinery give phenotypes in both basal and stimulated secretion, indicating that there is a single core exocytic machine, whereas deletion of components of the regulatory machinery give variable phenotypes, as described below.

## Core Exocytic Machinery

**Figure 2:**
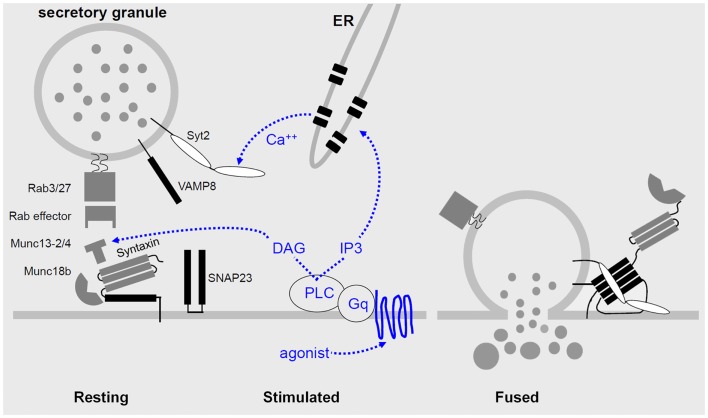
**Regulated airway mucin secretion**. Left – In the basal state, mucin granules are thought to become tethered to the plasma membrane by Rab proteins and effectors that have not yet been identified, in the vicinity of components of the exocytic machinery. Center – Activation of heptahelical receptors such as those for ATP (P_2_Y_2_) and adenosine (A3R) leads to activation of the trimeric G-protein, Gq, and phospholipase C (PLC), resulting in generation of the second messengers diacylglycerol (DAG) and inositol trisphosphate (IP_3_). Diacylglycerol activates the priming protein Munc13-2, and IP_3_ induces the release of calcium from apical ER to activate Synaptotagmin-2 (Syt2). Munc13-4 also participates in granule priming, and an unknown high affinity calcium sensor likely functions in basal secretion rather than Syt2. Right – Activation of the regulatory Munc13 and Syt proteins leads to full coiling of the SNARE proteins (SNAP23, VAMP8, and an unknown Syntaxin, all shown in black) to induce fusion of the granule and plasma membranes. The interactions of the SNARE proteins take place on a scaffold provided by Munc18b. In other secretory cells that form the basis for this model, exocytic Syntaxins contain four hydrophobic coiled-coil domains that must be opened to initiate secretion (left panel), and during fusion the associated Munc18 protein remains associated only by an interaction at the Syntaxin N-terminus (right panel).

Every step of vesicular transport on the exocytic and endocytic pathways involves the interactions of a four helix SNARE bundle with an SM protein (30, 54, 55). The SNARE proteins impart specificity to the pairing of transport vesicles with target membranes, mediate tight docking of vesicles to target membranes, and induce fusion of vesicle and target membranes when they fully coil. SM proteins provide an essential platform for sequential interactions of SNARE proteins, and also mediate interactions of the SNARE complex with tethering proteins (Figure 2). Three of the SNARE helices localize to the target membrane (called t-SNAREs for target SNAREs, or Q-SNAREs since the ionic amino acid of their SNARE domains is generally glutamine), and one SNARE helix is localized on the vesicle membrane (v-SNARE for vesicle SNARE, or R-SNARE since the ionic amino acid is generally arginine).

Syntaxins are Qa SNAREs that can be considered the central component of the core machinery since they initiate formation of the SNARE complex and their structure is ordered even in the absence of interaction with the other SNARE components. The Syntaxin that mediates mucin granule exocytosis remains unknown. Candidates are Stx 2, 3, and 11, all of which have been shown to functionally pair with Munc18b in other cell types since Munc18b has been definitively implicated in airway mucin exocytosis (56) (see below). Efforts are underway in the Dickey laboratory using genetically modified mice to test the roles of these Syntaxins in mucin secretion.

In both yeast and neurons, the Qb and Qc SNAREs involved in exocytosis are contributed by a single protein with two SNARE domains connected by a linker region. In yeast this protein is Sec9, which is essential for cell viability. In neurons the cognate protein mediating axonal synaptic vesicle release is SNAP25 (57). While SNAP25 is essential for post-natal life, brain development to the time of birth is nearly normal. In unpublished work, the Dickey laboratory has obtained evidence that SNAP23 mediates both basal and stimulated airway mucin secretion. SNAP23 is expressed ubiquitously, and knockout mice experience early embryonic lethality (58, 59). However heterozygous knockout mice show spontaneous airway epithelial cell mucin accumulation, indicating a defect in basal mucin secretion, as well as epithelial mucin retention after stimulation with aerosolized ATP, indicating a defect in stimulated secretion. Thus, SNAP23 appears to mediate most or all Qbc function in both basal and stimulated mucin secretion.

Recently, the R-SNARE (v-SNARE) in airway mucin secretion was identified as VAMP8 by immunolocalization to mucin secretory granules, *in vitro* functional analysis by RNA interference, and *in vivo* analysis of knockout mice (47). Both basal and stimulated mucin secretion were reduced by loss of VAMP8, though the defects were not as severe as from the loss of some other exocytic proteins, consistent with the viability of knockout mice, and suggesting that other v-SNAREs also participate in mucin secretion.

The scaffolding function of SM proteins in exocytosis in different cell types is mediated by three Munc18 proteins (54, 56). Munc18a (Stxbp1) and Munc18b (Stxbp2) appear to be paralogs functioning in axonal/apical secretion, whereas Munc18c (Stxbp3) is a ubiquitous isoform functioning in dendritic/basolateral secretion. Munc18a is expressed in neurons and neuroendocrine cells, whereas Munc18b is expressed in polarized epithelia. Together, these data suggested that Munc18b mediates airway mucin secretion, and localization and functional data support this. Munc18b is highly expressed in airway secretory cells where it localizes to the apical plasma membrane (56). Munc18b knockout mice are not viable postnatally, but heterozygous knockout mice show an ∼50% reduction in stimulated mucin secretion, indicating that Munc18b is a limiting component of the exocytic machinery (56). These heterozygous mutant mice do not show spontaneous mucin accumulation, unlike heterozygous SNAP23 mutant mice, suggesting that another SM protein besides Munc18b also plays a scaffolding role in basal mucin secretion whereas no other protein besides SNAP23 appears to function as a Qbc SNARE in mucin exocytosis. Ruling out the possibility that Munc18b functions only in stimulated and not basal mucin secretion, conditional mutant mice with Munc18b deleted only in airway secretory cells are viable and show spontaneous mucin accumulation, although preliminary results suggest that the accumulation is less than in Munc13-2 mice.

## Extracellular Signaling and the Exocytic Regulatory Machinery

The extracellular ligands and signal transduction pathways controlling mucin secretion have been studied for longer and in more depth than the exocytic machinery itself (30). The best-studied ligand is ATP that acts on the P_2_Y_2_ receptor to activate Gq and PLC-β1, resulting in generation of the second messengers IP_3_ and diacylglycerol (DAG). ATP is released in a paracrine fashion from ciliated cells in response to mechanical shear stress and in an autocrine fashion along with uridine nucleotides from secretory granules (60, 61). The ATP metabolite adenosine acting on the A3 adenosine receptor appears to activate the same Gq-PLC pathway (62). It is possible that other G-protein coupled receptors, such as those sensing serotonin or acetylcholine, also function on airway secretory cells since those ligands induce mucin secretion *in vivo* (34, 36), however they may be acting in a paracrine fashion by inducing contraction of smooth muscle cells leading to the release of ATP that in turn induces mucin release (Table 1).

In airway secretory cells, the second messenger IP_3_ activates receptors on apical ER to induce the release of calcium. In contrast to excitable cells in which calcium enters the cytoplasm from outside through voltage-gated channels, or secretory hematopoietic cells such as mast cells in which an initial release of calcium from intracellular stores triggers further calcium entry from outside through ICRAC, all of the cytoplasmic calcium involved in exocytic signaling in airway secretory cells appears to come from intracellular stores (30). This may be an adaptation to the fact that the calcium concentration in the thin layer of airway surface liquid is not stable due to the variable release of mucins that carry calcium as a counterion and the variable secretion via CFTR of bicarbonate that chelates calcium. Calcium does enter airway secretory cells from the basolateral surface to maintain intracellular stores, presumably by communication between the basolateral and apical ER since mitochondrial barriers segregate cytoplasmic calcium signals (63). Nonetheless, the chelation of extracellular calcium *in vitro* does not acutely affect mucin secretion. Rough ER at the apical pole of airway secretory cells lies in close apposition to mucin granules (46, 64), which should allow localized calcium signaling to the exocytic machinery through proteins such as Synaptotagmins and Munc13s.

Synaptotagmins are a family of proteins containing two C2 domains capable of calcium-dependent phospholipid binding, of which several members mediate calcium-dependent exocytosis. Using Syt2 knockout mice, we have found that Syt2 serves as a critical sensor of stimulated but not of basal mucin secretion (46). There was no spontaneous mucin accumulation in these mice, consistent with the fact that Syt2 and its close homolog Syt1 inhibit rather than promote synaptic vesicle release at baseline levels of cytoplasmic calcium (65). In contrast, there was a complete failure of ATP-stimulated mucin release in homozygous knockout mice and a dose-dependent failure in heterozygous knockout mice (46). This was a surprising result for several reasons. First, there is no impairment of synaptic vesicle release in heterozygous mutant Syt1 or Syt2 mice, indicating that some structural or functional feature of stimulated exocytosis in airway secretory cells differs from that in neurons to make Syt2 levels limiting, such as the difference in size of the secretory vesicles (50 nm in neurons versus 1000 nm in airway secretory cells) or the concentration of exocytic proteins at the active zone. Second, Syt2 is the fastest among the low affinity, fast calcium exocytic sensors Syt 1, 2, and 9, yet mucin secretion is a slow exocytic process (measured in hundreds of milliseconds) compared to synaptic vesicle release (measured in milliseconds). This suggests that some other feature of Syt2 besides its kinetics makes it a suitable regulator of mucin release. The calcium-sensing protein in basal mucin secretion that performs a role comparable to that of Syt2 in stimulated secretion is not yet known. Nonetheless such a protein likely exists since a second, high affinity calcium sensor functions in neurons and neuroendocrine cells, and basal mucin secretion has been shown to be calcium dependent (66).

Munc13 comprises a family of four calcium and lipid sensing proteins with variable numbers of C1 and C2 domains that function in the priming of secretory vesicles. As mentioned above, Munc13-2 knockout mice show defects in both basal and stimulated mucin secretion, with the basal defect being more dramatic than the stimulated defect (18). Munc13-2 contains a C1 domain that binds the second messenger DAG. Another member of this family, Munc13-4, is also expressed in airway secretory cells (67). Munc13-4 does not contain a C1 domain, though it does contain two C2 domains that may bind phospholipids in a calcium-dependent manner. In unpublished results, the Dickey laboratory has found that deletion of Munc13-4 causes a mild defect in stimulated secretion, and that deletion of both Munc13-2 and Munc13-4 together causes a severe (though incomplete) defect in stimulated secretion. Whether a third protein also functions in mucin granule priming to account for the residual secretion or whether mucin granule exocytosis depends only partially on priming function is not yet known.

There are additional targets of the regulation of mucin secretion besides Synaptotagmin and Munc13 proteins, such as PKC that also binds DAG and calcium (37, 38). Here we have focused on components of the exocytic machinery. A full accounting of the regulation of mucin secretion will require further knowledge of second messengers and their targets, together with analysis of their integrated function.

## Summary

Airway secretory cells continuously synthesize and secrete polymeric mucins that form a protective mucus layer. Both the synthesis and secretion of mucins are highly regulated, with low basal rates and high stimulated rates for each. Mature mucin granules are positioned for secretion by interactions of MARCKS, CSP, HSP70, Rab proteins, and the cytoskeleton. A core exocytic machine consisting of the SNARE proteins VAMP8, SNAP23, and an unknown Syntaxin, along with the scaffolding protein Munc18b, mediates both basal and stimulated mucin secretion. Regulatory proteins including Munc13-2, Munc13-4, and Syt2 respond to second messengers to control the rate of mucin secretion in response to extracellular signals. These regulatory proteins show differential activities in basal and stimulated secretion, suggesting that they variably associate with the core machinery depending on the levels of second messengers. Close coordination of mucin production and secretion with physiologic need are essential to lung health since either a deficiency or excess of airway mucus causes disease. The medical importance of airway mucin secretion and its scientific value as a model of large-granule exocytosis in polarized epithelia insure its continued study.

## Conflict of Interest Statement

Kenneth B. Adler has an ownership interest in BioMarck Pharmaceuticals that is developing the MANS peptide for commercial use. Michael J. Tuvim and Burton F. Dickey have no potential conflicts of interest.
